# Stress-induced remodeling of membrane-bound organelles in budding yeast

**DOI:** 10.1016/j.crmicr.2026.100653

**Published:** 2026-07-28

**Authors:** Peng Sheng, Mengna Sun, Xinyue Wang, Lizhe Bai, Hong Cao, Dan Li

**Affiliations:** a Department of Nutrition, Affiliated Hospital of Jiangnan University, Wuxi, China; b Wuxi School of Medicine, Jiangnan University, Wuxi, China; c Institute of Future Food Technology, JITRI, Yixing 214200, China

**Keywords:** Budding yeast, Organelle remodeling, Environmental stress, Fluorescence imaging, Mitochondria, Endoplasmic reticulum

## Abstract

Membrane-bound organelles undergo extensive remodeling during environmental stress, yet systematic side-by-side comparisons of organelle responses in budding yeast remain limited. Here, a panel of fluorescent markers was used to examine multiple organelles in *Saccharomyces cerevisiae* exposed to heat, hydrogen peroxide, acetic acid, or ethanol. Across all conditions, mitochondria consistently shifted from tubular networks to fragmented puncta, representing a common stress response. Quantitative scoring showed that heat stress induced mitochondrial fragmentation in more than 90% of cells within 20 min, and GFP-HDEL redistribution was detected in a substantial fraction of cells under all four stress conditions, with the strongest effect under heat stress. In contrast, overall endoplasmic reticulum (ER) morphology remained largely preserved, although redistribution of GFP-HDEL indicated altered ER retention and/or endomembrane homeostasis. Heat and oxidative stress also induced Ire1 puncta. Several nuclear proteins exhibited stress-dependent redistribution from the nucleus, indicating dynamic remodeling of nuclear protein localization. Vacuoles generally appeared enlarged and fused, whereas acetic acid induced a distinct phenotype with Ybh3 enrichment at the vacuolar membrane and redistribution of Prc1 and Pep4 to cytoplasmic puncta. Markers of the early and late Golgi and the late endosome showed stress-specific loss, clustering, or relocalization. Lipid droplets, peroxisomes, and autophagy-related structures were also altered. Additionally, Yca1, Aif1, and Mmi1 formed puncta under heat and ethanol stress. Together, these findings provide a comparative imaging framework defining shared and stress-specific features of organelle remodeling in budding yeast.

## Highlights

• Mitochondrial fragmentation is a uniersal stress reponse in budding yeast under heat, H_2_O_2_ and ethanol stress.
• Multiple membrane-bound organelles (ER, nucleus, vacuole, Golgi, endosomes, lipid droplets, peroxisomes) show stress-specific remodeling patterns.
• ER morphology remains largely intact, while GFP-HDEL redistribution indicates altered ER retention and disrupted endomembrane homeostasis under stress.
• Stress-related Yca1, Aif1 and Mmi1 form cytoplasmic puncta specifically upon heat and ethanol stress.
• This work establishes a systematic comparative imaging framework to study organelle dynamics in yeast under environmental stress.

## Graphical abstract

This graphical abstract summarizes our systematic analysis of organelle remodeling in Saccharomyces cerevisiae under four environmental stresses. Live-cell imaging reveals that mitochondrial fragmentation is a universal stress response, while other organelles and stress-related proteins exhibit stress-specific patterns of reorganization, providing a unified framework for understanding fungal stress adaptation.

**Figure:**
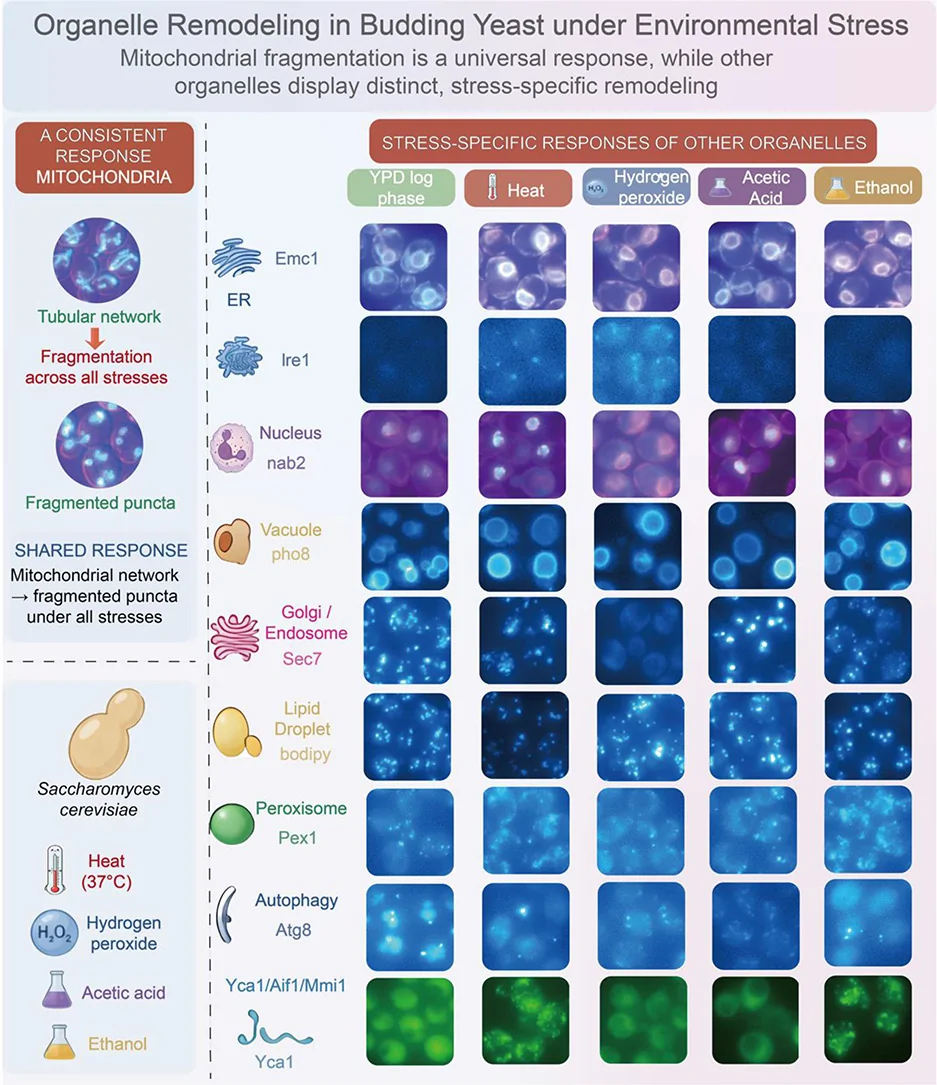

## 1 Introduction

*Saccharomyces cerevisiae* is widely used both as a model eukaryote and as an industrial cell factory. During laboratory cultivation and industrial fermentation, yeast cells frequently encounter heat stress, ethanol accumulation, weak organic acids, oxidative stress, and other dynamic environmental fluctuations, all of which can impair growth, reduce production performance, and disrupt intracellular homeostasis (Lin et al., 2022; Yuan et al., 2024; Kocik et al., 2026; Tartik, 2026). Because many adaptive and damage-control processes are organized at the level of intracellular compartments, understanding how organelles respond to environmental stress is important for both basic cell biology and the development of stress-tolerant strains. Membrane-bound organelles are central to stress sensing, adaptation, and cell fate control (Galluzzi et al., 2014). Mitochondria rapidly remodel during stress and are closely connected to redox balance and regulated cell death in yeast (Guaragnella et al., 2012; Ott et al., 2007). The endoplasmic reticulum (ER) coordinates protein folding and lipid biosynthesis, and ER perturbation activates the unfolded protein response and related stress pathways (Cebulski et al., 2011; Welihinda and Kaufman, 1996; Korennykh et al., 2009; Bartolutti et al., 2025; Mori, 2009). Correct ER retention and retrieval of luminal proteins are also essential for yeast growth and secretory homeostasis (Townsley et al., 1994). The vacuole contributes to degradation, ion storage, and stress adaptation, and acetic acid stress in yeast has been linked to vacuolar dysfunction and protease-dependent processes (Chaves et al., 2021; Ludovico et al., 2001; Saravanan and Kane, 2026). In parallel, the Golgi and endosomal systems organize cargo sorting and membrane traffic (Dean et al., 1997; Behnia et al., 2004; Trautwein et al., 2006; Franzusoff et al., 1992), whereas peroxisomes, lipid droplets, and autophagy-related structures support lipid metabolism, redox homeostasis, and autophagy-related quality control (Birschmann et al., 2005; Nair et al., 2012; Zhang et al., 2025; Li et al., 2026).

In addition to the autonomous responses of individual organelles, stress-induced organelle remodeling is increasingly recognized to involve membrane contact sites and inter-organelle communication. Membrane contact sites are specialized regions where two organelles are closely apposed without membrane fusion, allowing coordinated lipid exchange, metabolite transfer, ion homeostasis, membrane trafficking, and stress signaling (Phillips and Voeltz, 2016; Castro et al., 2022). In budding yeast, ER–mitochondria contacts mediated in part by the ERMES complex, mitochondria–vacuole contacts, nucleus–vacuole junctions, ER–vacuole contacts, and ER–endosomal contacts provide structural platforms for communication among organelles (Kornmann et al., 2009; Elbaz-Alon et al., 2014; Pan et al., 2000; Hariri et al., 2018; Hariri et al., 2016). These contact sites contribute to mitochondrial homeostasis, lipid metabolism, vacuolar function, membrane trafficking, autophagy, and adaptation to metabolic or environmental stress. Therefore, stress-dependent redistribution of organelle markers may reflect not only intrinsic remodeling of individual organelles, but also altered communication among organelles through dynamic membrane contact sites.

Several proteins previously linked to yeast cell death or stress adaptation also show stress-dependent changes in localization and function. The metacaspase Yca1 has functions extending beyond a simple pro-death role (Lam and Sherlock, 2023), the yeast Aif1 protein is an apoptosis-inducing factor orthologue (Wissing et al., 2004), and Mmi1 has been associated with stress granules in heat-shocked cells (Rinnerthaler et al., 2013). More broadly, recent views of the heat-shock response emphasize dynamic protein compartmentalization and condensate-like behavior rather than a strictly linear damage pathway (Dea and Pincus, 2024), and recent biochemical work further highlights the heterogeneous composition of yeast stress granule cores (Demeshkina and Ferré-D’Amaré, 2025). These observations suggest that stress-induced protein redistribution should be interpreted in the broader context of stress adaptation and proteostasis. Accordingly, the present study is intended primarily as a morphology- and localization-based survey, rather than a direct functional dissection of apoptosis, necrosis, or autophagic flux.

Although fluorescent-protein-based organelle marker collections and live-cell imaging protocols have been established for budding yeast (Zhu et al., 2019; Liu et al., 2022; Rines et al., 2011), and recent spatial proteomics has greatly expanded our understanding of stress-induced protein relocalization during ER stress (Platzek et al., 2025), several important questions remain unresolved. First, it remains unclear whether different environmental stresses induce a shared organelle-remodeling program or distinct compartment-specific responses. Second, organelle morphology and marker localization may be uncoupled, but these features have rarely been compared across multiple compartments in a unified framework. Third, single-cell imaging studies reveal substantial heterogeneity and incomplete penetrance of stress phenotypes, underscoring the need for quantitative scoring (Mattiazzi Usaj et al., 2020; Chong et al., 2015). These gaps motivated our comparative imaging analysis of organelle remodeling under heat, oxidative, acetic acid, and ethanol stress. In the present study, we used a panel of fluorescent organelle markers to examine the responses of mitochondria, the ER, the nucleus, the vacuole, the Golgi, endosomes, peroxisomes, lipid droplets, autophagy-related structures, and several stress-associated proteins in budding yeast. Rather than assigning each phenotype to a single mechanistic pathway, our goal was to define shared and stress-specific patterns of intracellular reorganization that may serve as a descriptive and hypothesis-generating framework for future mechanistic studies. By integrating multiple organelles, multiple stressors, and quantitative phenotype scoring within a unified imaging framework, this study establishes a comparative organelle-remodeling map that distinguishes broadly conserved responses, such as mitochondrial fragmentation, from more stress-dependent phenotypes, including GFP-HDEL redistribution, nuclear protein relocalization, acetic-acid-associated vacuolar remodeling, Golgi/endosomal marker redistribution, lipid droplet changes, peroxisome clustering, and GFP-Atg8 remodeling.

## 2 Materials and methods

### 2.1 Yeast strains, media and culture conditions

All strains used in this study were derived from *Saccharomyces cerevisiae* BY4741 (MATa his3Δ1 leu2Δ0 ura3Δ0 met15Δ0). The strains generated and used in this work are listed in Table 1. Cells were routinely cultured in yeast extract peptone dextrose (YPD) medium containing 1% (w/v) yeast extract, 2% (w/v) peptone, and 2% (w/v) D-glucose. Solid medium was prepared by supplementing YPD with 2% (w/v) agar.

**Table 1:** *Saccharomyces cerevisiae* strains used in this study.

Strain	Relevant genotype
BY4741	MATa his3Δ1 leu2Δ0 met15Δ0 ura3Δ0
YB1	BY4741 OM14::OM14-2GFP-URA3(K. lactis)
YB2	BY4741 CIT1::CIT1-2GFP-URA3(K. lactis)
YB3	BY4741 AFG3::AFG3-2GFP-URA3(K. lactis)
YB4	BY4741 ABF2::ABF2-2GFP-URA3(K. lactis)
YB5	BY4741 COX9::COX9-mTagBFP-kanMX NDI1::NDI1-2GFP-URA3(K. lactis)
YB6	BY4741 COX9::COX9-mTagBFP-kanMX NUC1::NUC1-2GFP-URA3(K. lactis)
YB7	BY4741 ELO3::ELO3-mTagBFP-kanMX EMC1::EMC1-2GFP-URA3(K. lactis)
YB8	BY4741 pTPI1::pTPI1-SS-GFP-HDEL-URA3(K. lactis)
YB9	BY4741 IRE1::IRE1-2GFP-URA3(K. lactis)
YB10	BY4741 ELO3::ELO3-mTagBFP-kanMX NAB2::NAB2-GFP-URA3(K. lactis)
YB11	BY4741 NSR1::NSR1-GFP-URA3(K. lactis)
YB12	BY4741 PUS1::PUS1-GFP-URA3(K. lactis)
YB13	BY4741 NHP6A::NHP6A-2GFP-URA3(K. lactis)
YB15	BY4741 NMA111::NMA111-2GFP-URA3(K. lactis)
YB17	BY4741 PHO8::pCU-GFP-PHO8-URA3(K. lactis)
YB18	BY4741 YBH3::YBH3-2GFP-URA3(K. lactis)
YB19	BY4741 PRC1::PRC1-2GFP-URA3(K. lactis)
YB20	BY4741 PEP4::PEP4-2GFP-URA3(K. lactis)
YB21	BY4741 CHS5::CHS5-GFP-URA3(K. lactis)
YB22	BY4741 SEC7::SEC7-2GFP-URA3(K. lactis)
YB23	BY4741 VRG4::VRG4-2GFP-URA3(K. lactis)
YB24	BY4741 SYS1::SYS1-2GFP-URA3(K. lactis)
YB25	BY4741 SNF7::SNF7-GFP-URA3(K. lactis)
YB26	BY4741 VPS4::VPS4-GFP-URA3(K. lactis)
YB27	BY4741 PEX1::PEX1-2GFP-URA3(K. lactis)
YB28	BY4741 ATG8::pATG8-GFP-ATG8-URA3(K. lactis)
YB29	BY4741 COX9::COX9-mTagBFP-kanMX YCA1::YCA1-2GFP-URA3(K. lactis)
YB30	BY4741 COX9::COX9-mTagBFP-kanMX AIF1::AIF1-2GFP-URA3(K. lactis)
YB31	BY4741 ELO3::ELO3-mTagBFP-kanMX MMI1::pMMI1-2GFP-MMI1-URA3(K. lactis)
YB32	BY4741 Hsp104::Hsp104-2GFP-URA3(K. lactis)
YB33	BY4741 Pab1::Pab1-2GFP-URA3(K. lactis)

For nitrogen starvation experiments, cells were transferred to synthetic dextrose nitrogen starvation (SD-N) medium containing 0.17% (w/v) yeast nitrogen base without amino acids and ammonium sulfate and 2% (w/v) D-glucose. For live-cell imaging, cells were washed and resuspended in synthetic minimal dextrose (SMD) medium containing 2% (w/v) glucose, 0.67% (w/v) yeast nitrogen base without amino acids, 30 mg l⁻¹ adenine, 30 mg l⁻^1^ lysine, 30 mg l⁻¹ methionine, 20 mg l⁻¹ histidine, 20 mg l⁻¹ uracil, 50 mg l⁻¹ tryptophan, and 50 mg l⁻¹ leucine. Unless otherwise indicated, cells were grown at 30 °C in YPD to mid-log phase (OD600 ≈ 0.7) before stress treatment or imaging.

### 2.2 Stress treatments

For heat-stress experiments, mid-log-phase cultures grown in YPD at 30 °C were shifted to 42 °C and incubated for the indicated times before imaging. For acetic acid stress, acetic acid was added directly to mid-log-phase cultures to the indicated final concentrations of 0.1% (v/v, 16.7 mM, pH 3.45), 0.2% (v/v, 33.4 mM, pH 3.27), 0.4% (v/v, 66.8 mM, pH 3.04), or 0.6% (v/v, 100.2 mM, pH 2.88). For ethanol stress, ethanol was added directly to mid-log-phase cultures to the indicated final concentrations of 2%, 4%, 8%, or 10% (v/v). For oxidative stress, H₂O₂ was added directly to mid-log-phase cultures to the indicated final concentrations of 0.5, 1.0, 2.0, or 4.0 mM. Cells were imaged after treatment for the durations specified in each experiment and figure legend. Heat-stress time-course experiments were imaged at 10, 20, 40, and 60 min after the shift to 42 °C, unless otherwise indicated. Ethanol, acetic acid, and H₂O₂ treatments were imaged after 1 h under the indicated conditions.

### 2.3 Plasmid construction and yeast strain generation

Some of the plasmids used in this study were derived from constructs reported in our previous work (Li et al., 2020), whereas the remaining plasmids were generated using standard molecular cloning procedures. In brief, DNA fragments were amplified by PCR, digested with the appropriate restriction enzymes, and ligated into the indicated vectors. Prior to yeast transformation, plasmids were linearized to direct integration at the intended genomic locus. Most fluorescent protein fusions were generated as C-terminal genomic integrations and were therefore expressed from their endogenous loci, unless otherwise indicated. In contrast, GFP-Pho8, GFP-Atg8, and 2GFP-Mmi1 were expressed as additional-copy constructs. Correct plasmid construction and genomic integration were verified by diagnostic PCR across the insert or integration junctions and by Sanger sequencing (Genewiz, China).

### 2.4 Rationale for organelle marker selection

The selected markers were chosen to cover major membrane-bound organelles and, where possible, distinct subcompartments or functional aspects of the same organelle. For mitochondria, we included markers labeling the outer membrane, matrix, mitochondrial DNA-associated proteins, respiratory-chain-associated proteins, and mitochondrial nucleoids to ensure that the observed fragmentation was not marker-specific. For ER, we used both membrane-associated ER markers and the luminal retention reporter GFP-HDEL. For Golgi and endosome, we selected early Golgi, late Golgi, and late endosome markers to distinguish different trafficking compartments. For autophagy and lipid droplets, GFP-Atg8 and BODIPY/TAG measurement were used to monitor autophagy-related structures and lipid storage, respectively. This marker panel was not intended to exhaustively cover all suborganellar domains, but to provide representative and orthogonal readouts for major membrane-bound organelles and selected stress-associated protein relocalization events.

The plasmids used in this study are listed in Table 2.

**Table 2:** Plasmids used in this study.

Plasmid	Backbone / parental plasmid	Integration or expression context
OM14-2GFP-ter-URA3	UG72-2GFP	C-terminal genomic integration
CIT1-2GFP-ter-URA3	UG72-2GFP	C-terminal genomic integration
AFG3-2GFP-ter-URA3	UG72-2GFP	C-terminal genomic integration
ABF2-2GFP-ter-URA3	UG72-2GFP	C-terminal genomic integration
COX9-mTagBFP-ter-kanMX	UG6-mTagBFP	C-terminal genomic integration
NDI1-2GFP-ter-URA3	UG72-2GFP	C-terminal genomic integration
NUC1-2GFP-ter-URA3	UG72-2GFP	C-terminal genomic integration
ELO3-mTagBFP-ter-kanMX	UG6-mTagBFP	C-terminal genomic integration
EMC1-2GFP-ter-URA3	UG72-2GFP	C-terminal genomic integration
pTPI1-SS-GFP-HDEL-URA3	UG72-GFP	TPI1 promoter-driven expression
IRE1-2GFP-ter-URA3	UG72-2GFP	C-terminal genomic integration
NAB2-GFP-ter-URA3	UG72-GFP	C-terminal genomic integration
NSR1-GFP-ter-URA3	UG72-GFP	C-terminal genomic integration
PUS1-2GFP-ter-URA3	UG72-2GFP	C-terminal genomic integration
NHP6A-2GFP-ter-URA3	UG72-2GFP	C-terminal genomic integration
NMA111-2GFP-ter-URA3	UG72-2GFP	C-terminal genomic integration
pCU-GFP-PHO8-ter-URA3	UG72-2GFP	Additional-copy expression from the PHO8 ORF context
YBH3-2GFP-ter-URA3	UG72-2GFP	C-terminal genomic integration
PRC1-2GFP-ter-URA3	UG72-2GFP	C-terminal genomic integration
PEP4-2GFP-ter-URA3	UG72-2GFP	C-terminal genomic integration
CHS5-GFP-ter-URA3	UG72-GFP	C-terminal genomic integration
SEC7-2GFP-ter-URA3	UG72-2GFP	C-terminal genomic integration
VRG4-2GFP-ter-URA3	UG72-2GFP	C-terminal genomic integration
SYS1-2GFP-ter-URA3	UG72-2GFP	C-terminal genomic integration
SNF7-GFP-ter-URA3	UG72-GFP	C-terminal genomic integration
VPS4-GFP-ter-URA3	UG72-GFP	C-terminal genomic integration
PEX1-2GFP-ter-URA3	UG72-2GFP	C-terminal genomic integration
pATG8-GFP-ATG8-ter-URA3	UG72-2GFP	ATG8 promoter-driven expression
YCA1-2GFP-ter-URA3	UG72-2GFP	C-terminal genomic integration
AIF1-2GFP-ter-URA3	UG72-2GFP	C-terminal genomic integration
pMMI1-2GFP-MMI1-ter-URA3	UG72-2GFP	Additional-copy expression

### 2.5 Live-cell fluorescence microscopy

For fluorescence microscopy, 1 ml of yeast culture was collected for each sample, washed once, and resuspended in SMD medium immediately before imaging. The brief washing and imaging procedure did not visibly alter the major stress-induced phenotypes analyzed in this study. Cell suspensions were loaded onto concanavalin A-coated 35-mm glass-bottom dishes and allowed to settle for 2 min. Fresh SMD medium was then added, and cells were imaged on an Olympus IX83 inverted fluorescence microscope equipped with a Hamamatsu ORCA-Flash4.0 LT camera and a Lumencor Spectra X six-channel light source. Images were acquired using a 100× oil-immersion objective lens. For routine imaging, z-stacks of 15 optical sections were acquired at 0.5 μm intervals. Unless otherwise indicated, exposure times were 100 ms per frame for GFP- and mTagBFP-labeled samples. Differential interference contrast (DIC) images were acquired in parallel where indicated. Within each experiment, image acquisition settings were kept constant across conditions and were adjusted during initial setup to avoid detector saturation in the displayed channels. All imaging experiments were performed in at least three independent biological replicates, and representative images are shown. For display, maximum-intensity projections were generated from z-stacks, whereas single optical sections were used for representative colocalization images. For puncta counting, the same thresholding method was applied across control and stress-treated samples within the same experiment. Mitochondria were scored as tubular when continuous elongated networks were visible and as fragmented when most mitochondrial signal appeared as discrete puncta or short discontinuous fragments. Nuclear markers were scored as redistributed when the nuclear signal was markedly reduced and/or the nuclear-to-cytoplasmic contrast was lost. GFP-HDEL was scored as redistributed when the signal was no longer predominantly ER-restricted and became diffuse or vacuole-associated. At least 100 cells per condition were scored in each biological replicate.

### 2.6 BODIPY staining of lipid droplets

Lipid droplets were visualized by BODIPY staining. Briefly, 1 μl of BODIPY 493/503 (Invitrogen, D3922) solution (0.01 mg ml⁻¹) was added to 100 μl of yeast culture. The stained cells were allowed to settle on concanavalin A-coated glass-bottom dishes for 2 min, washed with fresh SMD medium, and imaged after replacement with fresh SMD medium. Images were acquired using the same microscope described above with an exposure time of 100 ms.

### 2.7 MitoTracker staining

Yeast cells were incubated with MitoTracker (MitoTracker™ Green FM, Thermo Fisher, M7514) at a final concentration of 1 μM for 3 min. After staining, cells were washed once with fresh medium and resuspended for live-cell imaging. MitoTracker fluorescence was predominantly localized to mitochondria and was used to assess mitochondrial morphology under control and stress conditions.

### 2.8 FM4-64 staining

Yeast cells were cultured in YPD liquid medium until the optical density at 600 nm (OD) reached 0.7 for subsequent staining experiments. The lipophilic fluorescent dye FM4-64 (Beyotime Biotechnology, Cat. No. C1889S) was added to the yeast culture at a final dilution ratio of 2500:1, and the cells were continuously incubated for 20 min at 30 °C for pulse labeling. After pulse incubation, the cell samples were collected for microscopic observation. Subsequently, the stained cells were harvested by centrifugation, washed once with sterile phosphate-buffered saline (PBS), and resuspended in fresh prewarmed YPD medium for chase incubation. For stress and temperature treatment assays, the resuspended yeast cells were divided into different groups. One group was cultured at 30 °C with the addition of acetic acid, hydrogen peroxide, and ethanol, while another group was incubated at 42 °C for heat stress treatment. FM4-64 fluorescence signals of all experimental groups were observed and recorded by fluorescence microscopy at 20 min and 1 h post-treatment, respectively.

### 2.9 Biochemical quantification of triacylglycerol

Cellular triacylglycerol (TAG) content was quantified using a commercial TAG assay kit (E1013, Applygen Technologies, Beijing, China) based on the glycerol phosphate oxidase (GPO) enzymatic method. For sample preparation, yeast cell cultures with an optical density (OD) of 0.8 were collected, and a total of 4 mL of the cell suspension (equivalent to 4 OD units) was harvested for each sample. The yeast cells were pelleted via centrifugation at 12,000 rpm for 1 min at room temperature. The cell pellets were then subjected to one freeze–thaw cycle to fully disrupt cell structures, followed by digestion with yeast lyticase for 30 min to release intracellular lipids. Subsequent TAG quantification was strictly performed in accordance with the manufacturer’s standard protocols. Finally, the measured TAG content was normalized to the total protein content of each sample to eliminate differences in cell loading and obtain the relative cellular TAG level.

### 2.10 Image processing and quantification

Image processing and quantitative analysis were performed using Fiji/ImageJ (ImageJ version 1.54p; Fiji version 1.16.0). Images within the same experiment were acquired using identical imaging settings and processed consistently. Brightness and contrast were adjusted linearly and identically across groups for presentation purposes only and did not affect quantitative analysis. Maximum-intensity projections generated from 15-slice z-stacks spanning 7 μm with a 0.5-μm interval were used for morphological assessment unless otherwise specified. Single optical sections through the central cellular plane were used only for representative visualization of spatial overlap; no quantitative colocalization analysis was performed.

For phenotype quantification, at least 100 cells per condition were scored in each biological replicate from at least three independent fields using predefined criteria. Mitochondria were classified as fragmented when the network consisted predominantly of short, discontinuous, or punctate structures, whereas mitochondria with predominantly elongated and interconnected networks were classified as tubular. Nuclear and ER signals were classified according to their retention, marked reduction, or redistribution, and punctate and diffuse patterns were distinguished based on their intracellular distribution. Images were coded before analysis, and scoring was performed with the investigator blinded to the experimental conditions.

For whole-cell fluorescence measurements, individual cell boundaries were manually outlined as regions of interest (ROIs) based on the corresponding DIC images. Because no independent nuclear marker was used, nuclear ROIs were manually defined according to the boundaries of the nuclear fluorescence signal, using the same predefined ROI-selection criteria across all experimental groups. At least 30 cells per condition were analyzed in each biological replicate. For each ROI, the background-corrected mean fluorescence intensity was calculated as the mean ROI intensity minus the mean local background intensity measured in a cell-free region of the same image. The same ROI-selection, background-subtraction, and measurement procedures were applied consistently across all experimental groups. At least three independent biological replicates were analyzed, with the biological replicate used as the unit of statistical analysis.

### 2.11 Western blot analysis of yeast cell proteins

Total yeast proteins were extracted via modified TCA precipitation. Yeast pellets were resuspended in lysis buffer and lysed by glass bead vortexing. Clarified supernatants were quantified by BCA assay and equalized for SDS-PAGE prior to Western blotting with anti-GFP antibody (M20004S, Abmart). Since heat, hydrogen peroxide, acetic acid and ethanol treatments perturb endogenous GAPDH levels, total protein from Coomassie Brilliant Blue-stained gels served as the loading control. Post-electrophoresis gel staining was performed, and grayscale values of total protein bands were quantified to normalize GFP band intensities.

### 2.12 Statistical analysis

All quantitative data are presented as mean ± SD from at least three biologically independent experiments; the exact n and the quantified parameter for each panel are indicated in the corresponding figure legend. Comparisons between two groups were performed using an unpaired two-tailed Student’s t-test, whereas comparisons among multiple groups were analyzed by one-way ANOVA followed by Tukey’s multiple-comparison test. A *P* value < 0.05 was considered statistically significant. All quantitative analyses were performed using data from independent biological replicates; individual cells within the same image field were not treated as independent biological observations for statistical testing.

## 3 Results

### 3.1 Mitochondria

**Fig. 1:**
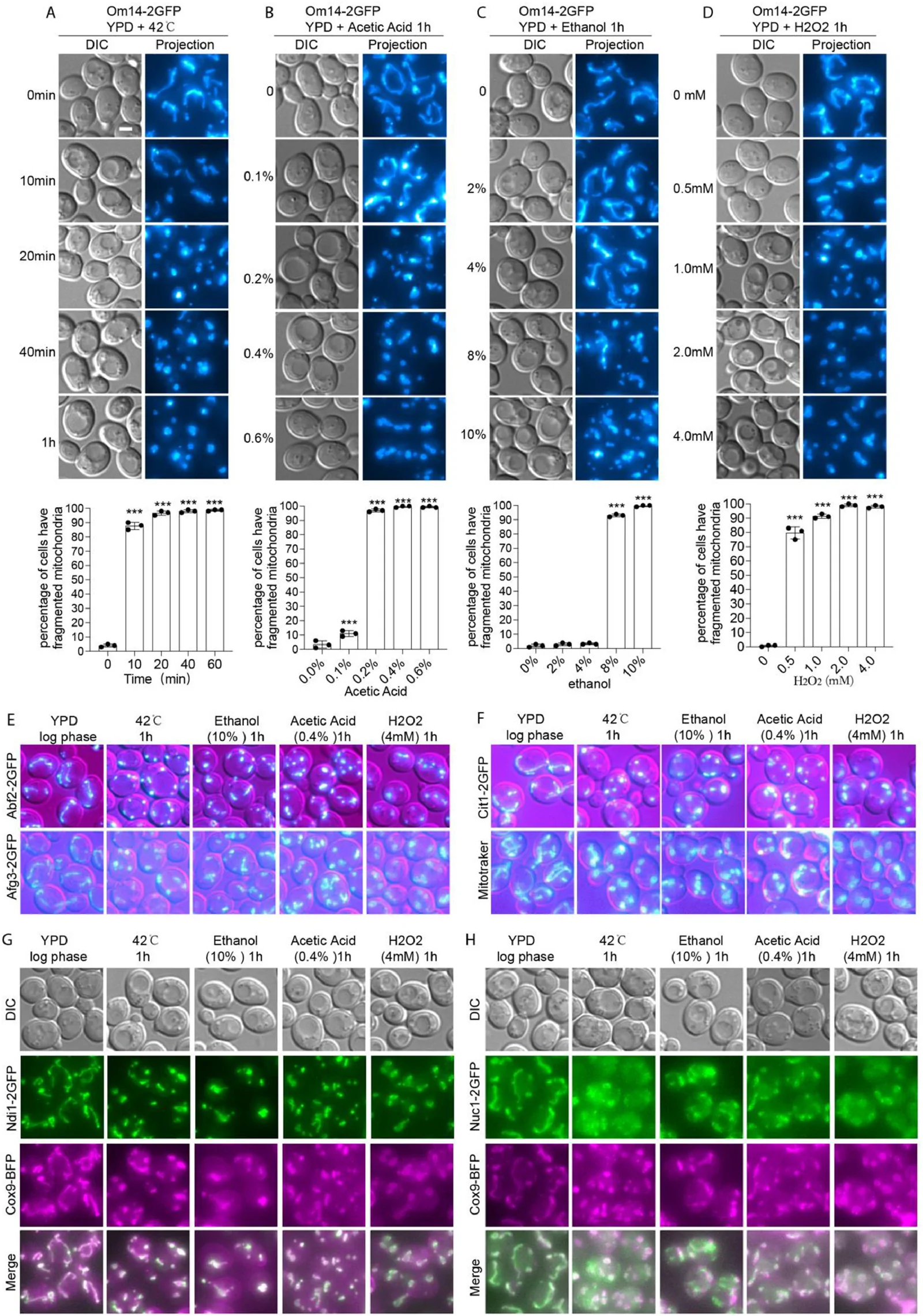
**Stress-triggered mitochondrial remodeling in budding yeast**. Mid-log-phase yeast cells expressing the indicated mitochondrial reporters were exposed to heat, acetic acid, ethanol, or H₂O₂ and analyzed by live-cell fluorescence microscopy. Z-stacks were acquired as 15 optical sections at 0.5 μm intervals; maximum-intensity projections are shown for morphology analysis, whereas single optical sections are shown for colocalization analysis. (A–D) Representative Om14-2GFP images and quantification of cells with fragmented mitochondria under the indicated stresses. (E, F) Validation with additional mitochondrial markers, including Abf2-2GFP, Afg3-2GFP, Cit1-2GFP, and MitoTracker staining. (G, H) Colocalization analysis of Ndi1-2GFP or Nuc1-2GFP with Cox9-mTagBFP. Maximum-intensity projections are shown for morphology analysis, and single optical sections are shown for colocalization analysis. Scale bar, 2 μm. Data are mean ± SD from three independent biological replicates; at least 100 cells were scored per condition per replicate. Statistical significance was determined by one-way ANOVA followed by Tukey’s multiple-comparison test. *P < 0.05, **P < 0.01, ***P < 0.001.

To assess stress-induced changes in mitochondrial morphology, we monitored mitochondria using multiple fluorescent markers, including Om14-2GFP, Abf2-2GFP, Afg3-2GFP, Cit1-2GFP, MitoTracker, Cox9-mTagBFP, Ndi1-2GFP, and Nuc1-2GFP, selected from a previously validated organelle marker toolkit (Zhu et al., 2019). Phenotypes were interpreted using a simplified morphology classification framework that distinguishes tubular networks from fragmented/punctate mitochondrial structures. Under logarithmic growth in YPD medium at 30 °C, all of these markers revealed the typical tubular mitochondrial network (Fig. 1A–H). After a shift from 30 °C to 42 °C, mitochondrial tubules began to fragment within 5 min and were almost completely converted into punctate structures after 20 min (Fig. 1A). Time-course quantification revealed a rapid and progressive increase in mitochondrial fragmentation after the heat shift, with fragmentation detectable at the earliest time point and exceeding 90% of cells by 20 min (Fig. 1A). Similar fragmentation was observed under acetic acid, ethanol, and H₂O₂ stress (Fig. 1B–D and Fig. S1A–D). In particular, filamentous mitochondria were largely lost at the higher concentrations of acetic acid (0.6%) and ethanol (10%) tested in this study (Fig. 1B, C). Comparable changes were observed with additional genetically encoded mitochondrial markers and with MitoTracker staining, supporting the conclusion that the stress-induced transition from tubular networks to fragmented or punctate structures was a robust mitochondrial remodeling response rather than a reporter-specific artifact (Fig. 1A–H).

We next examined whether stress altered the localization of two mitochondria-associated proteins previously linked to cell death. Ndi1-2GFP remained colocalized with Cox9-mTagBFP under heat, acetic acid, ethanol, and H₂O₂ stress (Fig. 1G). Likewise, Nuc1-2GFP remained colocalized with Cox9-mTagBFP under all four conditions (Fig. 1H). Thus, although mitochondrial morphology was strongly remodeled by stress, Ndi1 and Nuc1 remained associated with mitochondria under the conditions tested.

### 3.2 Endoplasmic reticulum (ER)

**Fig. 2:**
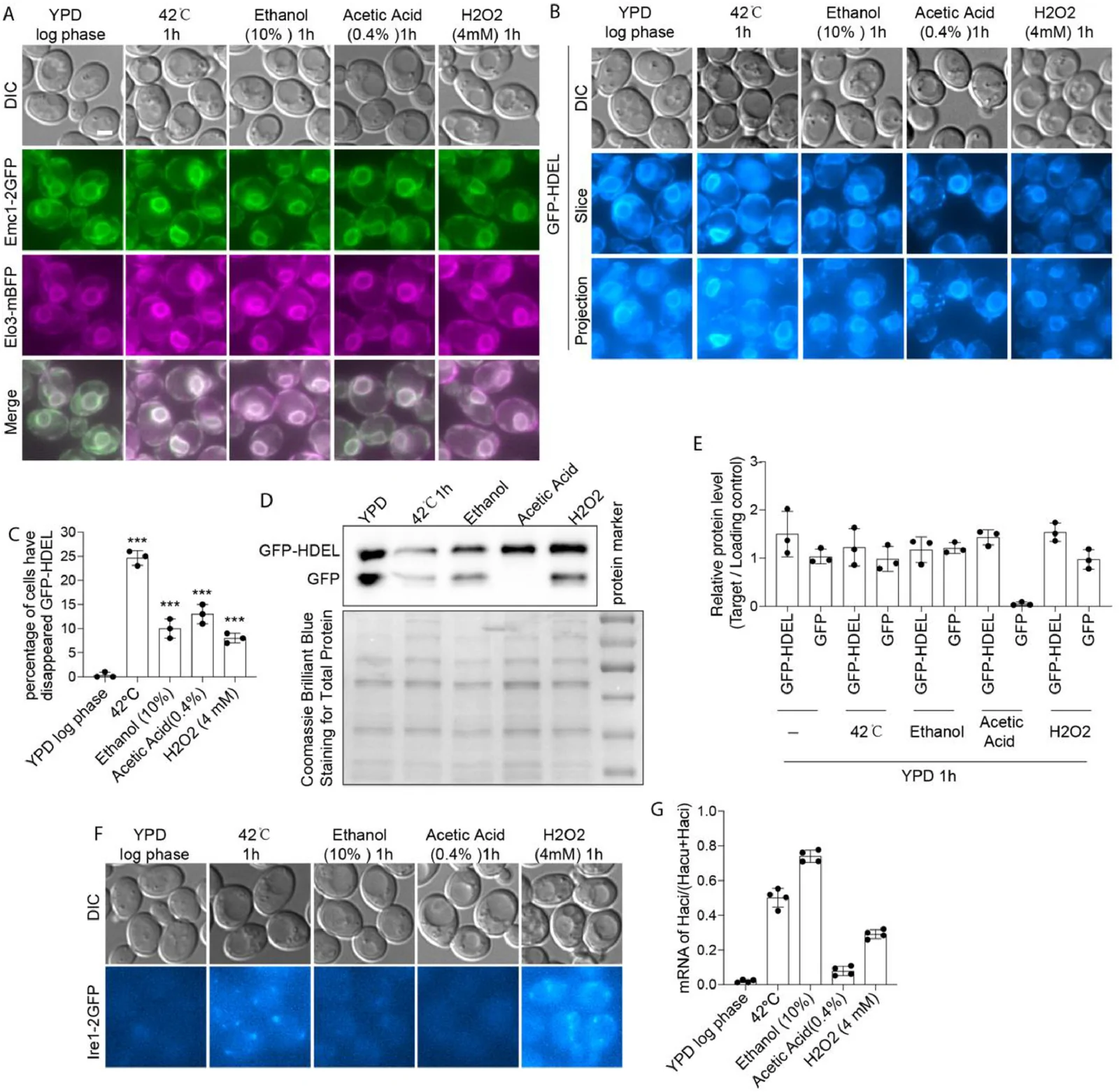
**Stress responses of the endoplasmic reticulum.** Images were acquired as described in Fig. 1. (A) Representative images of ER architecture labeled by Emc1-2GFP and Elo3-mTagBFP. (B, C) Representative images and quantification of stress-induced GFP-HDEL redistribution. (D, E) Western blot analysis and densitometric quantification of full-length GFP-HDEL and free GFP, normalized to total cellular protein. (F) Representative images of Ire1-2GFP puncta formation. (G) qRT-PCR analysis of the HAC1i/HAC1u mRNA ratio as a readout of canonical Ire1-HAC1 UPR activation. Scale bar, 2 μm. Data are presented as mean ± SD from three independent biological replicates; at least 100 cells were scored per condition per replicate for cell-based quantification. Statistical significance was determined by one-way ANOVA followed by Tukey’s multiple-comparison test. *P < 0.05, **P < 0.01, ***P < 0.001.

To assess ER responses, we examined cells expressing Emc1-2GFP, Elo3-mTagBFP, or GFP-HDEL. Under logarithmic growth conditions, all three markers labeled the characteristic ER network, including cortical ER, perinuclear ER, and connecting ER structures (Fig. 2A, B). Under heat, acetic acid, ethanol, and H₂O₂ stress, the overall ER morphology visualized by Emc1-2GFP and Elo3-mTagBFP remained largely preserved, and both markers continued to colocalize (Fig. 2A). In contrast, GFP-HDEL showed marked redistribution under all four stress conditions, with the strongest effect observed during heat stress (Fig. 2B, C). Rather than remaining confined to the ER, the signal became diffused in the cytoplasm and/or accumulated in vacuole-associated structures, suggesting stress-dependent impairment of ER luminal protein retention/retrieval and broader endomembrane trafficking homeostasis. To further validate the stress-induced alterations in GFP-HDEL distribution, we performed Western blotting (WB) to assess the intracellular abundance and cleavage of GFP-HDEL under the four tested stress conditions (Fig. 2D, E). WB results showed that total GFP-HDEL protein levels were moderately decreased under heat stress. Moreover, free GFP accumulation was reduced in cells treated with heat, ethanol, and H₂O₂. Notably, free GFP was nearly undetectable following acetic acid exposure, which is consistent with reduced GFP stability and/or enhanced degradation under acidic stress conditions, rather than a simple redistribution of intact GFP-HDEL. Collectively, these biochemical results support the microscopic observations and demonstrate that different stressors differentially perturb GFP-HDEL protein stability, ER luminal protein retention, and endomembrane homeostasis, with heat and acetic acid producing the most prominent effects on GFP-HDEL behavior.

We also examined Ire1-2GFP as a marker of ER stress-associated puncta formation. Heat stress and H₂O₂ induced clear Ire1 puncta, whereas acetic acid and ethanol did not produce comparable Ire1 clustering under the conditions tested (Fig. 2F). To further assess the differential activation of the canonical Ire1-HAC1 unfolded protein response pathway, we quantified the relative levels of spliced and unspliced HAC1 transcripts (HAC1i and HAC1u) by quantitative real-time PCR (qRT-PCR) (Fig. 2G). Transcriptional analysis showed that heat stress, ethanol, and H₂O₂ significantly increased the relative abundance of spliced HAC1 and/or the HAC1i/HAC1u ratio. In contrast, acetic acid treatment had no obvious effect on canonical HAC1 splicing under the same experimental conditions. These results suggest that GFP-HDEL redistribution and canonical Ire1-HAC1 UPR activation can be partially uncoupled under environmental stress, especially under acetic acid treatment.

### 3.3 Nucleus

**Fig. 3:**
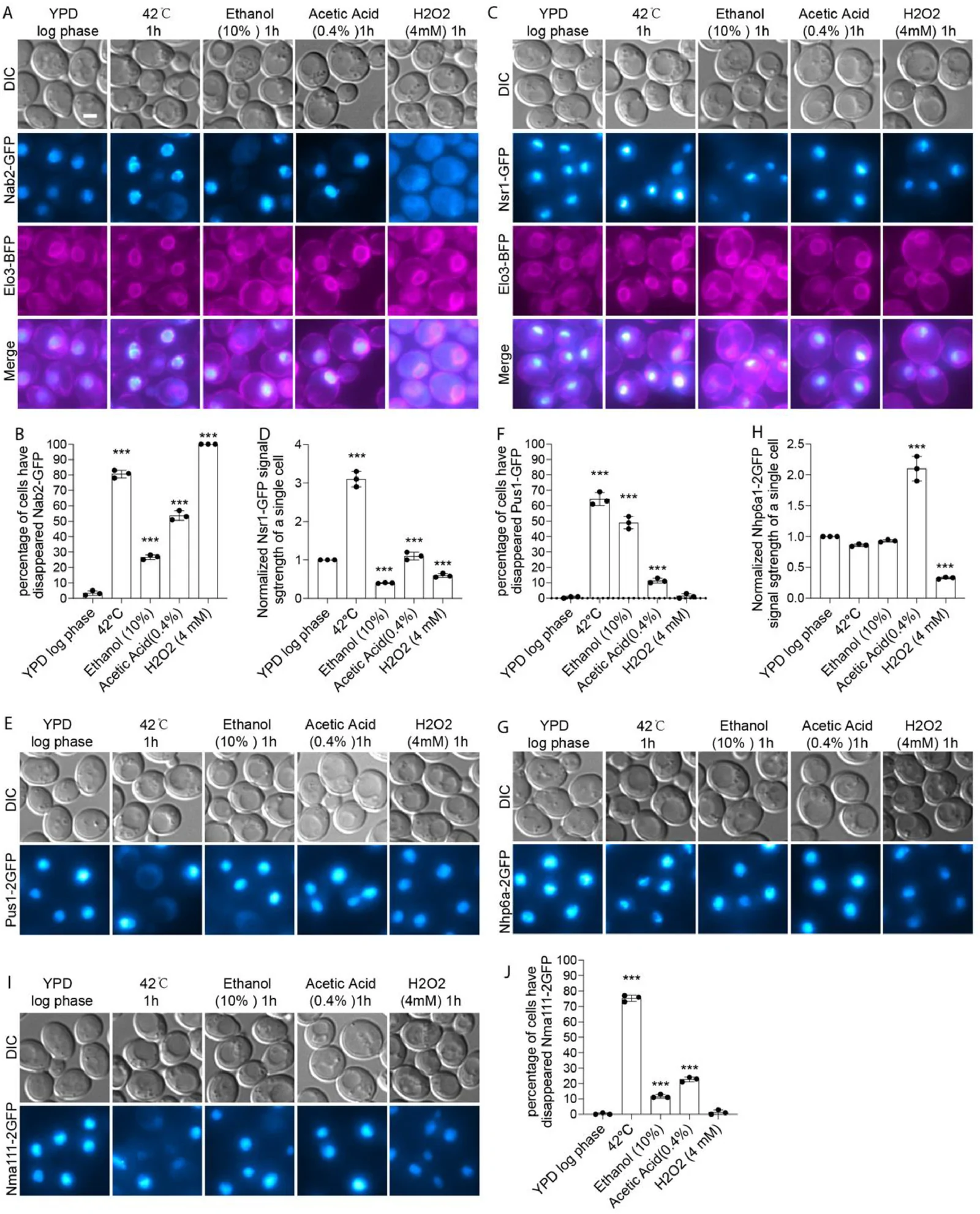
**Stress-induced redistribution of nuclear proteins.** Images were acquired as described in Fig. 1. (A, C, E, G, I) Representative images of Nab2-GFP, Nsr1-GFP, Pus1-2GFP, Nhp6a-2GFP, and Nma111-2GFP under control and stress conditions. (B, F, J) Quantification of cells showing markedly reduced nuclear signals for Nab2-GFP, Pus1-2GFP, and Nma111-2GFP. (D, H) Quantification of normalized single-cell fluorescence intensities for Nsr1-GFP and Nhp6a-2GFP. Where indicated, Elo3-mTagBFP was used as a reference marker for cellular morphology. Scale bar, 2 μm. Data are mean ± SD from three independent biological replicates; at least 100 cells were scored per condition per replicate for cell-based quantification. Statistical significance was determined by one-way ANOVA followed by Tukey’s multiple-comparison test. *P < 0.05, **P < 0.01, ***P < 0.001.

To characterize stress-triggered nuclear remodeling, we tracked the subcellular distribution of five nuclear reporters: Nab2-GFP, Nsr1-GFP, Pus1-2GFP, Nhp6a-2GFP, and Nma111-2GFP. Under log-phase YPD culture, all five fusion proteins predominantly localized to the nucleus, providing distinct readouts of nuclear or nucleolar protein localization (Fig. 3A–J).

Heat treatment at 42 °C induced pronounced marker-specific nuclear remodeling. Nab2-GFP, Pus1-2GFP, and Nma111-2GFP showed markedly reduced nuclear signals, with more than 60% of cells displaying diminished nuclear enrichment for these reporters (Fig. 3B, F, J). Consistently, single-cell fluorescence measurements showed significant reductions in Pus1-2GFP and Nma111-2GFP signals (Fig. S2A–C). By contrast, Nsr1-GFP displayed increased nuclear/nucleolar signal intensity, whereas Nhp6a-2GFP showed only minor changes in fluorescence intensity after heat exposure (Fig. 3D, H).

Ethanol stress similarly reduced the nuclear enrichment of Nab2-GFP and Pus1-2GFP, accompanied by significant decreases in the fluorescence intensity of Pus1-2GFP and Nma111-2GFP. The remaining reporters showed comparatively mild localization changes under ethanol treatment (Fig. 3A–J and Fig. S2A–C). Acetic acid treatment reduced the nuclear signals of Nab2-GFP, Pus1-2GFP, and Nma111-2GFP relative to unstressed log-phase cells, while Nhp6a-2GFP showed a pronounced increase in nuclear fluorescence intensity (Fig. 3H). H₂O₂ exposure caused strong depletion of nuclear Nab2-GFP, together with moderate attenuation or redistribution of the other nuclear GFP reporters (Fig. 3A–J).

Collectively, these data demonstrate that diverse environmental stresses elicit stress- and protein-specific changes in nuclear reporter localization and fluorescence intensity, rather than a uniform nuclear response across all tested nuclear factors. These observations further suggest that nuclear remodeling under stress involves selective redistribution or altered detectability of specific nuclear proteins, rather than a generalized loss of nuclear organization.

### 3.4 Vacuoles

**Fig. 4:**
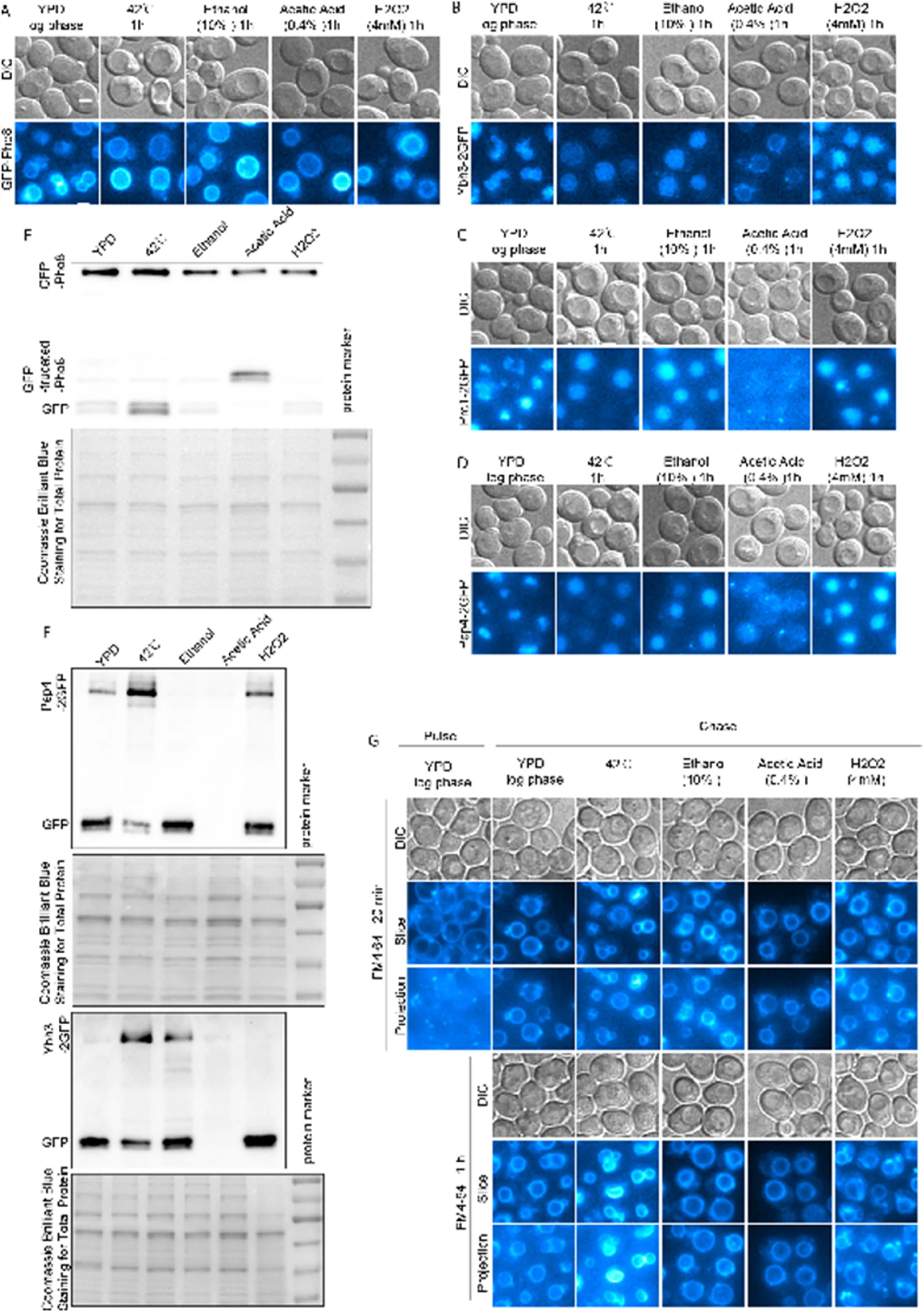
**Vacuolar remodeling under diverse environmental stresses.** Images were acquired as described in Fig. 1. (A–D) Representative images of GFP-Pho8, Ybh3-2GFP, Prc1-2GFP, and Pep4-2GFP under control and stress conditions. (E, F) Western blot analysis of GFP-Pho8, Pep4-2GFP, and Ybh3-2GFP processing; Coomassie Brilliant Blue staining was used as the total protein loading control. (G) FM4-64 pulse-chase imaging of vacuolar membrane labeling and endocytic trafficking at 20 min and 1 h after stress exposure. Scale bar, 2 μm. Data are representative of three independent biological replicates.

We next examined vacuolar responses using GFP-Pho8, Ybh3-2GFP, Prc1-2GFP, and Pep4-2GFP. Under logarithmic growth conditions, all four markers predominantly labeled vacuolar structures, with GFP-Pho8 showing prominent vacuolar membrane labeling (Fig. 4A–D). Under heat, ethanol, and H₂O₂ stress, vacuoles generally appeared enlarged and more fused, whereas the localization patterns of Ybh3-2GFP, Prc1-2GFP, and Pep4-2GFP were largely retained (Fig. 4A–D). In contrast, acetic acid induced a distinct vacuolar phenotype. Under this condition, Ybh3-2GFP redistributed predominantly to the vacuolar membrane (Fig. 4B). At the same time, Prc1-2GFP and Pep4-2GFP lost their typical vacuolar localization and appeared as one or a few cytoplasmic puncta in many cells (Fig. 4C, D). GFP-Pho8 remained detectable under the same condition, suggesting that the acetic-acid-induced phenotype could not be explained solely by a complete loss of GFP fluorescence.

To further evaluate stress-dependent changes in vacuolar GFP-tagged reporters, we performed Western blot analysis of GFP-Pho8, Pep4-2GFP, and Ybh3-2GFP, using Coomassie Brilliant Blue staining as the total protein loading control. These reporters showed distinct stress-dependent processing and stability patterns. For GFP-Pho8, full-length protein dominated under log-phase YPD conditions, with minimal free GFP cleavage. Heat stress promoted the accumulation of free GFP, whereas acetic acid induced additional truncated GFP-Pho8 species; ethanol and H₂O₂ caused only modest changes in GFP-Pho8 processing (Fig. 4E). For Pep4-2GFP, intact protein and free GFP were detected under control conditions. Ethanol and acetic acid markedly reduced full-length Pep4-2GFP, but their cleavage patterns differed: ethanol was associated with strong free GFP accumulation, whereas acetic acid produced little free GFP (Fig. 4F). For Ybh3-2GFP, full-length protein was weak under log-phase conditions, whereas free GFP was abundant. Heat and ethanol increased intact Ybh3-2GFP and reduced free GFP, while acetic acid depleted full-length Ybh3-2GFP with minimal cleavage products; H₂O₂ increased free GFP without detectable intact Ybh3-2GFP (Fig. 4F). Together, these biochemical data indicate that vacuolar GFP-tagged proteins undergo stress-specific changes in stability and proteolytic processing, which may contribute to the fluorescence patterns observed by microscopy.

Because GFP fluorescence can be affected by acidic and protease-rich vacuolar environments, especially under acetic acid stress, the Ybh3-, Prc1-, and Pep4-based imaging phenotypes should be interpreted cautiously. Thus, these data support the conclusion that acetic acid induces a distinct vacuolar remodeling phenotype, but they do not by themselves distinguish altered protein localization from changes in GFP stability, proteolysis, or fluorescence detectability.

To complement the GFP-based reporters, we performed FM4-64 pulse-chase labeling to monitor vacuolar membrane labeling and endocytic trafficking to the vacuole. After FM4-64 pulse labeling, cells were chased for 20 min or 1 h under log-phase control conditions or under heat, ethanol, acetic acid, or H₂O₂ stress. FM4-64 reached vacuolar membranes in all treatment groups at both chase time points, indicating that delivery of endocytic membrane tracer to vacuoles was not completely blocked under these conditions (Fig. 4G). However, distinct stress-specific FM4-64 labeling patterns were observed. Heat stress increased vacuolar FM4-64 signal intensity, whereas acetic acid substantially attenuated the vacuolar signal. Ethanol and H₂O₂ induced prominent accumulation of FM4-64-positive vesicular structures around vacuoles, with more pronounced vesicle clustering under H₂O₂ stress (Fig. 4G). Collectively, these observations suggest that environmental stresses differentially affect vacuolar morphology and endolysosomal membrane trafficking patterns, with acetic acid producing the most distinctive vacuolar reporter phenotype among the tested stressors.

### 3.5 Golgi and endosome

**Fig. 5:**
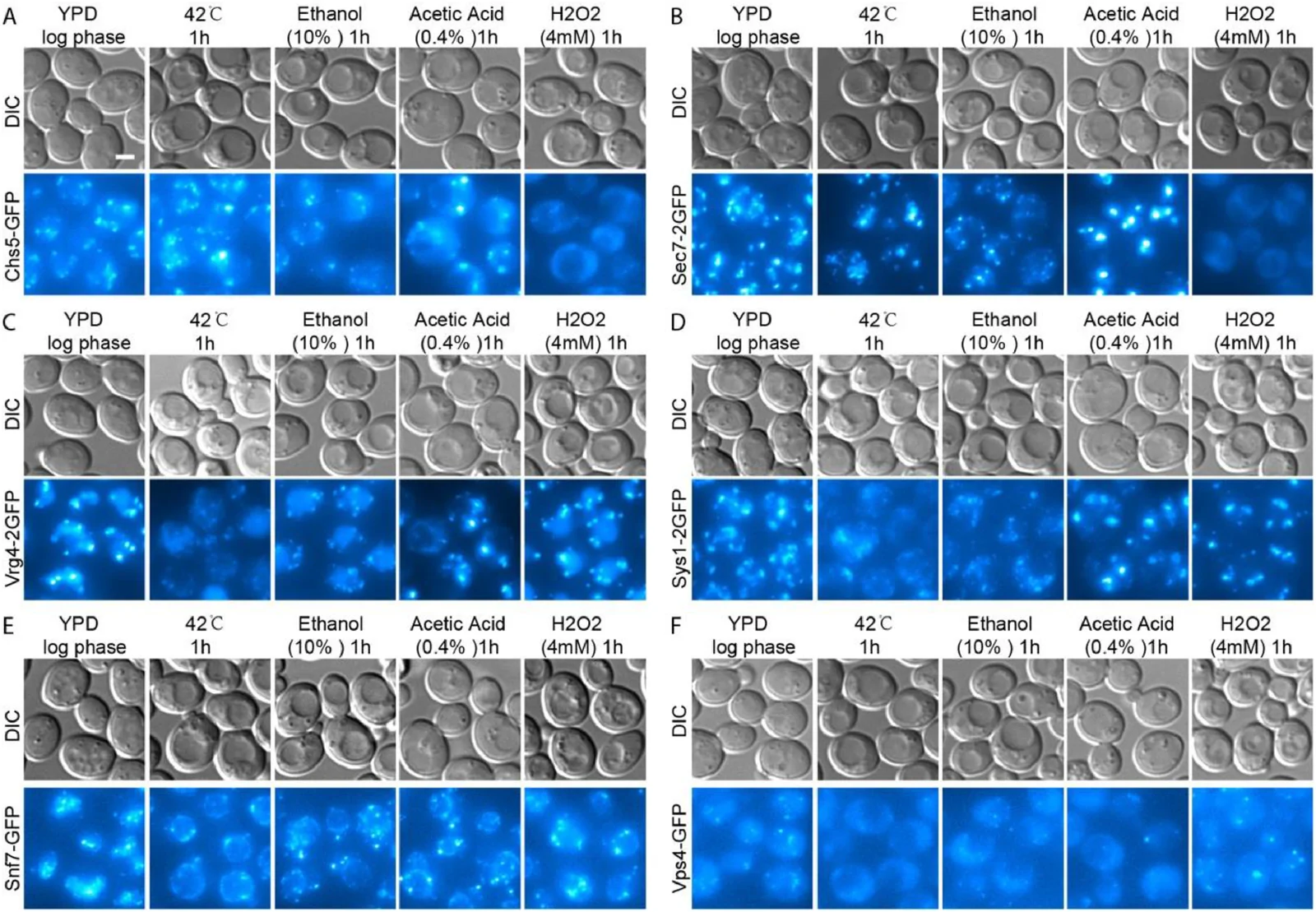
**Stress-induced remodeling of the Golgi and late endosome.** Images were acquired as described in Fig. 1. (A, B) Representative images of the Golgi-associated markers Vrg4-2GFP and Sys1-2GFP. (C, D) Representative images of Chs5-GFP and Sec7-2GFP. (E, F) Representative images of the late-endosome-associated markers Snf7-GFP and Vps4-GFP. Scale bar, 2 μm. Images are representative of three independent biological replicates and document stress-dependent redistribution patterns of Golgi- and late-endosome-associated markers.

To assess stress-induced remodeling of the secretory and endosomal systems, we analyzed the Golgi-associated markers Vrg4-2GFP and Sys1-2GFP, the late Golgi/trans-Golgi-associated markers Chs5-GFP and Sec7-2GFP, and the late-endosome/ESCRT-associated markers Snf7-GFP and Vps4-GFP. Under logarithmic growth conditions, these markers displayed their characteristic punctate distributions (Fig. 5A–F). Under heat and ethanol stress, the punctate signals of the early Golgi markers Vrg4-2GFP and Sys1-2GFP were strongly reduced (Fig. 5A, B). Under acetic acid and H₂O₂ stress, the number of Vrg4-2GFP- and Sys1-2GFP-positive puncta decreased, although the extent of signal loss varied among stress conditions (Fig. 5A, B).

Late Golgi markers showed additional stress-specific phenotypes. Under H₂O₂ stress, Chs5-GFP and Sec7-2GFP signals were markedly reduced (Fig. 5C, D). Under heat stress, the late Golgi puncta became clustered, whereas under ethanol stress the signals were markedly weakened (Fig. 5C, D). Under acetic acid stress, late Golgi puncta became enlarged and dimmer, with the effect being especially evident for Sec7-2GFP (Fig. 5C, D). These observations suggest that distinct stressors differentially affect Golgi-associated marker organization rather than producing a single uniform Golgi phenotype.

The two late endosome markers responded differently from one another. Snf7-GFP, which appeared as puncta under logarithmic growth conditions, showed prominent vacuole-associated redistribution under all four stress conditions (Fig. 5E). By contrast, Vps4-GFP signal was strongly reduced under heat, ethanol, and acetic acid stress, and was less affected under H₂O₂ stress (Fig. 5F). Because Snf7 is an ESCRT-III component whereas Vps4 is an AAA ATPase required for ESCRT-III remodeling and recycling, their differential behavior may reflect stress-dependent effects on distinct steps of late endosomal ESCRT dynamics. However, because tagged endomembrane markers such as Snf7 can themselves influence compartment morphology or function, these observations should be interpreted as marker-reported redistribution patterns within this imaging framework. These data indicate that environmental stress induces pronounced remodeling of Golgi- and endosome-associated marker patterns, with distinct reporters showing different sensitivities and redistribution behaviors.

### 3.6 Lipid droplets, peroxisomes, and autophagy-related structures

**Fig. 6:**
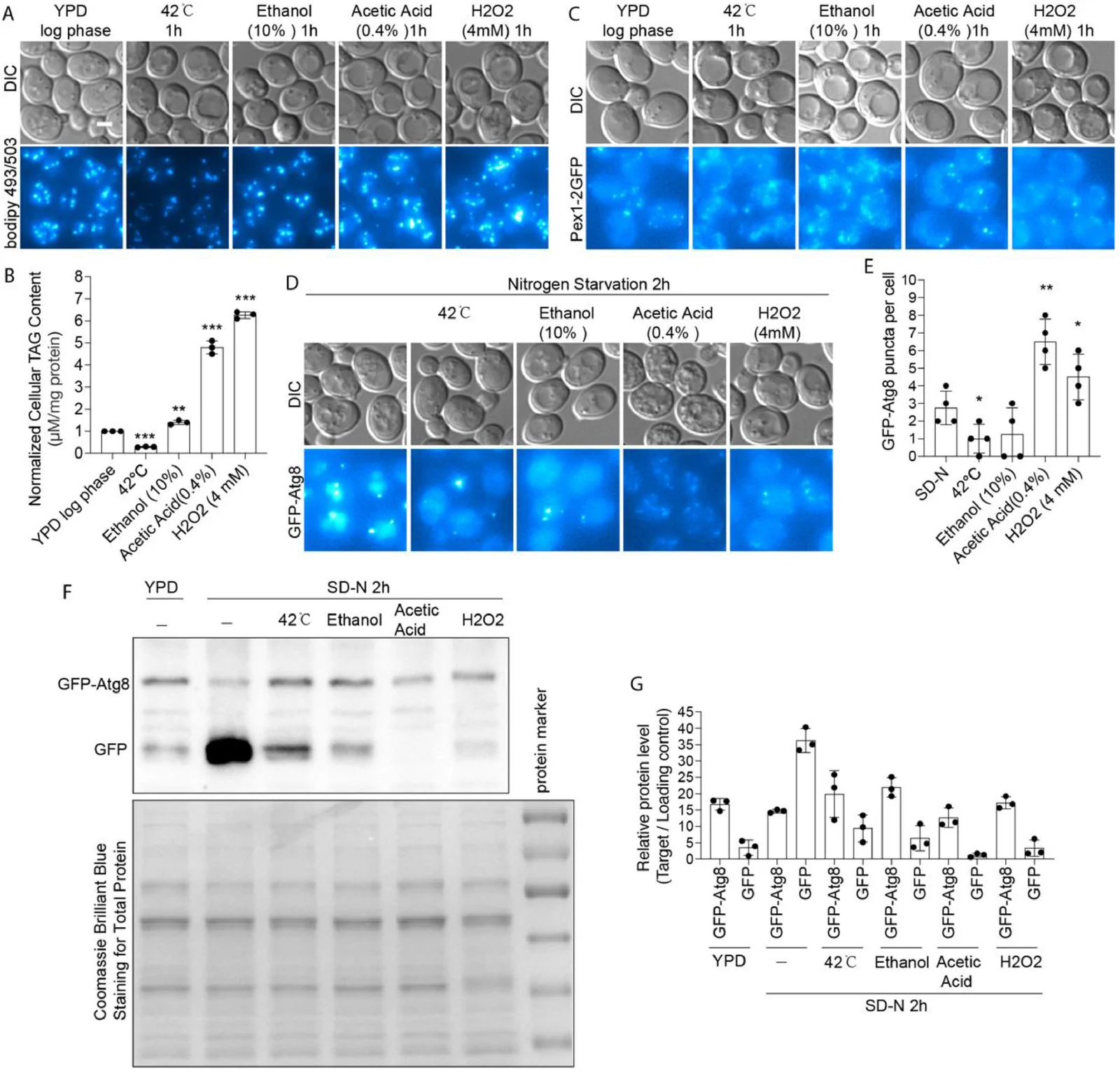
**Stress-induced remodeling of lipid droplets, peroxisomes, and autophagy-related structures.** Imaging parameters matched those described in Fig. 1. (A) Representative images of BODIPY-stained lipid droplets. (B) Quantification of total triacylglycerol (TAG) levels normalized to total cellular protein. (C) Representative images of Pex1-2GFP-labeled peroxisome-associated structures. (D, E) Representative GFP-Atg8 images and quantification of cells with prominent GFP-Atg8 puncta. (F, G) GFP-Atg8 cleavage immunoblot and densitometric quantification of full-length GFP-Atg8 and free GFP. Nitrogen starvation for 2 h was used as a positive control for autophagy induction. Scale bar, 2 μm. Data are mean ± SD from three independent biological replicates; at least 100 cells were scored per condition per replicate for cell-based quantification. Statistical significance was determined by one-way ANOVA followed by Tukey’s multiple-comparison test. *P < 0.05, **P < 0.01, ***P < 0.001.

We next examined lipid droplets by BODIPY staining and biochemical triacylglycerol (TAG) measurements. Heat stress markedly reduced the abundance of BODIPY-positive lipid droplets (Fig. 6A, B). In contrast, acetic acid and H₂O₂ promoted lipid droplet accumulation and/or clustering, whereas ethanol had a comparatively modest effect (Fig. 6A, B). Consistent with these imaging observations, TAG quantification provided a biochemical readout of stress-dependent changes in lipid storage. These results indicate that lipid storage compartments respond differentially to distinct stressors.

Peroxisomes were analyzed using Pex1-2GFP. Under acetic acid and H₂O₂ stress, Pex1-2GFP puncta remained largely similar to those in logarithmically growing cells (Fig. 6C). By contrast, heat stress and ethanol induced clustering of Pex1-2GFP puncta (Fig. 6C), indicating that peroxisome-associated marker organization was selectively altered under these conditions.

To assess autophagy-related structures, we examined GFP-Atg8. Under acetic acid and H₂O₂ stress, the number of GFP-Atg8-positive autophagy-related structures increased markedly (Fig. 6D, E). Under heat and ethanol stress, GFP-Atg8 signal became more diffuse and was redistributed from discrete Atg8-labeled structures and vacuole-associated signal to the cytoplasm (Fig. 6D, E). Thus, all four stress conditions altered GFP-Atg8 localization, although the resulting patterns differed substantially among stressors. Because GFP-Atg8 puncta formation does not necessarily correspond to increased autophagic flux, we further assessed autophagy using the GFP-Atg8 cleavage assay. Autophagic flux was evaluated by GFP-Atg8 cleavage immunoblotting under nutrient-replete conditions (YPD), nitrogen starvation (SD-N, 2 h), and nitrogen starvation combined with each of the four stress treatments (Fig. 6F, G). Comparable full-length GFP-Atg8 levels were detected in YPD-grown control cells and SD-N-starved cells without additional stressors. Robust free GFP accumulation was observed in nitrogen-starved yeast, consistent with active autophagic flux. After co-treatment with nitrogen starvation and heat, ethanol, acetic acid, or H₂O₂, free GFP signals were attenuated relative to the SD-N-only group. Among the tested stressors, heat exposure caused the mildest reduction in free GFP release, whereas acetic acid produced the strongest inhibition of GFP-Atg8 processing. Quantitative densitometric analysis supported these immunoblot observations, showing higher relative full-length GFP-Atg8 levels and reduced free GFP abundance in all stress co-treatment groups compared with nitrogen starvation alone. Collectively, these data indicate that under the tested co-treatment conditions, heat, ethanol, acetic acid, and H₂O₂ attenuate starvation-induced GFP-Atg8 processing to different degrees, suggesting stress-specific modulation of autophagic flux.

### 3.7 Stress-associated proteins

**Fig. 7:**
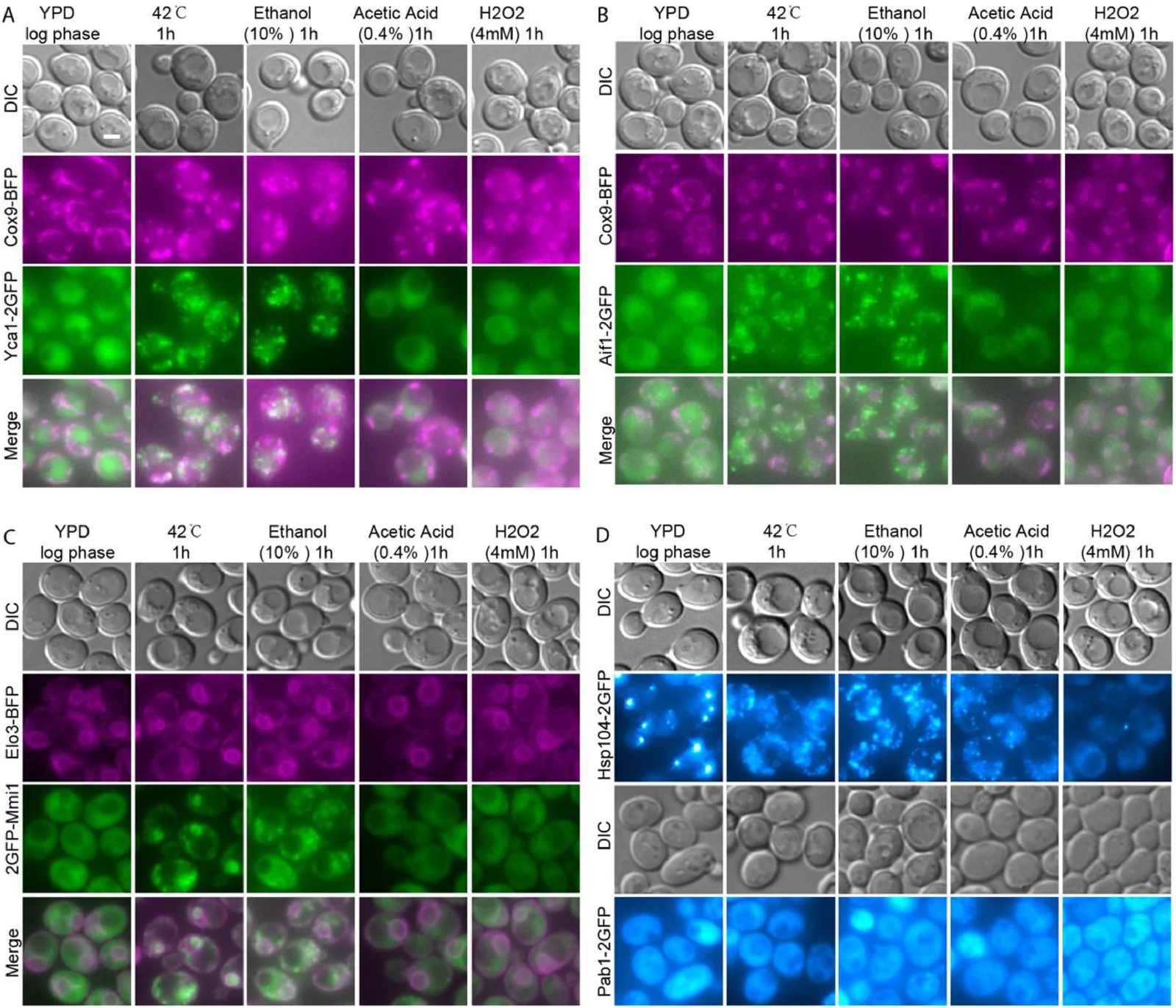
**Stress-triggered redistribution of stress-associated proteins.** Live-cell imaging was performed as detailed in Fig. 1. (A, B) Colocalization analysis of Yca1-2GFP or Aif1-2GFP with the mitochondrial marker Cox9-mTagBFP. (C) Colocalization analysis of 2GFP-Mmi1 with Elo3-mTagBFP as a cellular morphology reference marker. (D) Representative images of Hsp104-2GFP and Pab1-2GFP. Scale bar, 2 μm. Images are representative of three independent biological replicates.

Finally, we examined the localization of three stress-associated proteins previously implicated in stress adaptation and stress-associated phenotypes in yeast: Yca1-2GFP, Aif1-2GFP, and 2GFP-Mmi1. Under heat and ethanol stress, Yca1-2GFP formed cytoplasmic puncta instead of remaining diffusely distributed (Fig. 7A). These puncta did not colocalize with the mitochondrial marker Cox9-mTagBFP (Fig. 7A). Under acetic acid and H₂O₂ stress, the localization of Yca1-2GFP changed much less prominently (Fig. 7A).

Aif1-2GFP showed a similar pattern. Heat and ethanol induced punctate redistribution, whereas acetic acid and H₂O₂ caused less obvious changes, and the puncta observed under heat and ethanol did not colocalize with mitochondria (Fig. 7B). The behavior of 2GFP-Mmi1 also differed by stress condition. Heat and ethanol both induced Mmi1-positive puncta, but the heat-induced puncta remained partially associated with the nucleus, whereas the ethanol-induced puncta did not show obvious nuclear localization (Fig. 7C). Because Mmi1 has previously been linked to stress granules in heat-shocked yeast, these data suggest that Mmi1 redistribution may reflect stress-dependent protein compartmentalization rather than a uniform relocalization response across all stressors.

To provide reference markers for stress-induced protein compartmentalization, we additionally imaged Hsp104-2GFP and Pab1-2GFP (Fig. 7D). Hsp104-2GFP was used as a marker of protein aggregate/proteostasis-related structures, whereas Pab1-2GFP was used as a stress granule-associated reporter. Under YPD control conditions, Hsp104-2GFP displayed detectable punctate signals, indicating the presence of basal Hsp104-labeled protein quality-control structures. Heat, acetic acid, and H₂O₂ treatments reduced Hsp104-2GFP punctate signals, with the most pronounced loss observed under H₂O₂ stress, where Hsp104-positive puncta were nearly abolished. In contrast, ethanol treatment increased the number of Hsp104-2GFP puncta, although the fluorescence intensity of individual puncta appeared weaker. Pab1-2GFP showed a distinct stress-response pattern. Compared with YPD-grown cells, heat treatment did not cause an obvious increase in Pab1-2GFP puncta. Ethanol and acetic acid induced weak Pab1-2GFP punctate signals, with acetic acid producing a higher proportion of puncta-positive cells than ethanol. H₂O₂ treatment markedly enhanced the overall Pab1-2GFP fluorescence signal.

Together, these results indicate that Hsp104- and Pab1-labeled structures respond differently to distinct stressors. Thus, stress-induced protein compartmentalization in this system is marker- and stress-type-specific, rather than a uniform aggregation or stress granule response. These reference-marker patterns also caution against directly equating Yca1-, Aif1-, or Mmi1-positive puncta with canonical stress granules without additional colocalization or biochemical validation.

## 4 Discussion

This study provides a comparative imaging-based analysis of organelle remodeling in budding yeast under four common environmental stress conditions: heat, oxidative stress, ethanol, and acetic acid. Rather than focusing on a single compartment or a single stressor, our work offers a side-by-side view of how multiple intracellular structures respond to distinct stress inputs. Overall, our data suggest that organelle remodeling in yeast is both coordinated and stress-specific, with some responses broadly shared across conditions and others tightly linked to the nature of the stressor. This broader view is relevant to both the fundamental biology of yeast stress adaptation and efforts to engineer robust industrial strains (Lin et al., 2022; Yuan et al., 2024; Hariri et al., 2016). Importantly, the present study is intended primarily as a comparative imaging resource and descriptive framework, rather than a comprehensive mechanistic dissection of stress signaling, membrane trafficking, autophagic flux, or cell death pathways. In this respect, our work complements recent spatial proteomics studies, which have revealed extensive protein localization changes during ER stress at a proteome-wide scale (Platzek et al., 2025). Whereas spatial proteomics provides broad, unbiased information on protein relocalization, our live-cell imaging approach preserves single-cell morphology, organelle structure, marker-specific phenotypes, and cell-to-cell variability across multiple stress conditions. Thus, these two approaches provide complementary perspectives on stress-induced intracellular reorganization.

A common feature across all four stresses was mitochondrial fragmentation. Using multiple mitochondrial markers, we consistently observed a transition from a tubular network to punctate structures, indicating that mitochondrial remodeling is among the most robust morphological responses to stress in yeast in our imaging framework. This finding is consistent with previous work linking mitochondrial dynamics to stress adaptation and cell-death-associated phenotypes in yeast (Guaragnella et al., 2012; Ott et al., 2007; Lam and Sherlock, 2023). However, despite pronounced fragmentation, Ndi1 and Nuc1 remained associated with mitochondria in our experiments. This differs from reports describing stress-associated release or relocalization of Ndi1 and other mitochondrial factors under selected death-inducing conditions (Wissing et al., 2004), suggesting that mitochondrial fragmentation can occur without obligatory redistribution of these proteins in every stress context. Therefore, mitochondrial fragmentation should be interpreted as a robust stress-associated remodeling phenotype, but not by itself as direct evidence of mitochondrial outer membrane permeabilization or execution of a defined cell-death pathway.

The ER followed a different pattern. The overall ER morphology visualized by Emc1-2GFP and Elo3-mTagBFP remained largely preserved, suggesting that acute stress does not necessarily cause gross ER collapse in this system. However, GFP-HDEL redistributed markedly, especially under heat stress, pointing to altered ER retention and/or endomembrane trafficking rather than wholesale collapse of ER structure. The GFP-HDEL phenotype may reflect several non-mutually exclusive mechanisms. HDEL-containing luminal ER proteins are normally retrieved from the Golgi to the ER through the ERD2/HDEL receptor system (Townsley et al., 1994). Therefore, stress-induced GFP-HDEL redistribution may result from impaired ER retrieval, altered ER-to-Golgi cycling, changes in luminal protein retention, vacuolar delivery of mislocalized luminal proteins, or stress-dependent changes in GFP stability. The observation that ER membrane markers remained largely preserved while GFP-HDEL redistributed suggests that ER morphology and ER luminal protein retention can be partially uncoupled during stress. Accordingly, the GFP-HDEL phenotype is best viewed as a stress-associated redistribution pattern rather than a direct readout of a single trafficking defect. In addition, heat stress and H₂O₂ induced Ire1 puncta, consistent with previous reports linking Ire1 clustering to ER stress (Welihinda and Kaufman, 1996; Korennykh et al., 2009). Recent work further shows that ER stress in yeast drives broad proteome relocalization (Platzek et al., 2025), whereas UPR deficiency has major consequences for proteostasis and cellular fitness (Bartolutti et al., 2025; Mori, 2009). Taken together, these observations indicate that the four stressors do not perturb ER homeostasis in equivalent ways, and that gross ER morphology and ER functional output should not be assumed to change in parallel.

The nucleus also displayed pronounced marker-specific remodeling. Several nuclear proteins partially or completely redistributed from the nucleus under stress, but the magnitude and direction of change differed by marker and condition. Such differences argue against a simple, nonspecific breakdown of nuclear integrity and instead point to selective remodeling of nuclear protein homeostasis and nucleocytoplasmic partitioning. Because the markers used here represent different nuclear or nucleolar processes, the observed changes may reflect regulated nuclear transport, altered protein stability, stress-induced changes in nuclear organization, or changes in fluorescence detectability. Future work should determine whether these responses are linked to specific nuclear quality-control mechanisms, transcriptional adaptation, RNA metabolism, or stress-dependent remodeling of nucleocytoplasmic transport.

Among the organelles analyzed, the vacuole showed both a shared response and a stress-specific response. Under multiple stresses, vacuoles appeared enlarged and fused, which is consistent with adaptive remodeling of the lytic compartment during stress. In contrast, acetic acid produced a qualitatively distinct phenotype characterized by vacuolar membrane enrichment of Ybh3 and redistribution of Prc1 and Pep4 to cytoplasmic puncta. Acetic acid-induced death pathways in yeast have previously been linked to vacuolar proteases and vacuole-associated remodeling (Chaves et al., 2021; Ludovico et al., 2001; Rinnerthaler et al., 2013). Recent work further highlights the vacuole as an active stress-responsive organelle whose membrane remodeling and signaling functions can change rapidly during environmental challenge (Saravanan and Kane, 2026). However, because the identity of the observed puncta remains unresolved and membrane permeabilization was not directly measured here, our data are best interpreted as evidence of stress-specific vacuolar reorganization rather than as definitive support for a particular death mechanism. This cautious interpretation is further supported by the pH sensitivity and proteolytic vulnerability of GFP-based reporters in acidic vacuolar environments. Therefore, the acetic-acid-induced changes in Ybh3-, Prc1-, and Pep4-based signals may reflect a combination of true protein relocalization, altered proteolytic processing, GFP stability changes, and fluorescence detectability. Future validation with pH-stable fluorophores or complementary biochemical assays will be useful to distinguish true relocalization from fluorescence changes caused by vacuolar acidification.

The secretory and endosomal systems were also highly sensitive to environmental challenge. Early Golgi markers lost puncta or decreased in number, late Golgi markers showed clustering or fading, and the late endosome markers Snf7 and Vps4 responded differently from one another. These observations support the idea that membrane traffic is reorganized in a compartment-specific manner during stress rather than through uniform disassembly of the entire secretory pathway (Dean et al., 1997; Behnia et al., 2004; Trautwein et al., 2006; Franzusoff et al., 1992). The differential behavior of Snf7-GFP and Vps4-GFP is particularly informative. Snf7 is a core ESCRT-III component that assembles on endosomal membranes, whereas Vps4 is an AAA ATPase required for ESCRT-III remodeling, disassembly, and recycling (Babst et al., 2002; Azmi et al., 2006). Therefore, stress-induced redistribution of Snf7-GFP to vacuole-associated structures, together with reduced Vps4-GFP signal under selected stresses, may reflect stress-dependent effects on distinct steps of late endosomal ESCRT dynamics, including ESCRT-III membrane association, complex turnover, or recycling. More broadly, recent spatial proteomics has reinforced the view that stress-induced protein relocalization is a major and underappreciated component of yeast cellular reorganization (Platzek et al., 2025). The phenotype of GFP-HDEL and several Golgi markers is also consistent with the idea that endomembrane homeostasis is particularly vulnerable to acute stress (Townsley et al., 1994; Hariri et al., 2016; Li et al., 2020). Nevertheless, because the present data are based mainly on fluorescent marker redistribution, these observations should not be overinterpreted as direct evidence for a specific trafficking block without additional cargo transport, secretion, or ESCRT activity assays.

Changes in lipid droplets, peroxisomes, and autophagy-related structures further highlight the breadth of intracellular remodeling. Heat reduced lipid droplet abundance, whereas acetic acid and oxidative stress promoted lipid droplet accumulation or clustering, implying substantial rewiring of lipid handling under different conditions. Peroxisomes were comparatively resistant to acetic acid and H₂O₂ in our assay but aggregated under heat and ethanol stress. GFP-Atg8-labeled autophagy-related structures were altered under all four stresses, yet the resulting patterns were not equivalent. Because Atg8 localization alone cannot distinguish increased autophagosome formation from impaired autophagic flux (Nair et al., 2012; Zhang et al., 2025; Li et al., 2026), our data should be interpreted as evidence of autophagy-related remodeling rather than definitive inhibition or activation of autophagy. The GFP-Atg8 cleavage assay further indicated that, under the tested co-treatment conditions, heat, ethanol, acetic acid, and H₂O₂ attenuated starvation-induced GFP-Atg8 processing to different degrees. This result suggests that stress-induced GFP-Atg8 puncta formation and autophagic flux can be partially uncoupled, reinforcing the need to combine imaging readouts with biochemical flux assays.

The behavior of Yca1, Aif1, and Mmi1 indicates that stress also reshapes the localization of proteins often discussed in the context of yeast cell death. Recent work has emphasized that Yca1 has diverse functions extending beyond a simple apoptotic effector role (Lam and Sherlock, 2023), that Aif1 is best understood within specific stress contexts rather than as a universal death marker (Wissing et al., 2004), and that Mmi1 can associate with stress granules during heat shock (Rinnerthaler et al., 2013). In this light, the puncta observed here under heat and ethanol stress may reflect stress-induced compartmentalization, proteostasis-associated sequestration, or condensate-like behavior rather than cell death execution per se (Dea and Pincus, 2024; Demeshkina and Ferré-D’Amaré, 2025). The inclusion of Hsp104-2GFP and Pab1-2GFP further supports a cautious interpretation. Hsp104 is associated with protein disaggregation and proteostasis-related structures, whereas Pab1 is a commonly used stress granule-associated reporter in budding yeast (Yoo et al., 2022). In our experiments, Hsp104- and Pab1-labeled structures did not respond identically across stress conditions, indicating that stress-induced protein compartmentalization is marker- and stress-type-specific rather than a uniform aggregation response. Therefore, Yca1-, Aif1-, or Mmi1-positive puncta should not be equated with canonical stress granules or apoptotic structures without direct colocalization, biochemical fractionation, or functional validation. This interpretation is more consistent with the descriptive scope of the present dataset.

The main advance of this study is the establishment of a unified, side-by-side imaging framework that compares many organelles and stress-associated proteins under the same set of environmental stresses. Previous yeast organelle imaging resources and stress-response studies have provided essential marker collections, single-organelle observations, or global protein localization datasets, but fewer studies have systematically compared shared and stress-specific remodeling patterns across mitochondria, ER, nucleus, vacuole, Golgi/endosome, lipid droplets, peroxisomes, autophagy-related structures, and stress-associated proteins within one experimental design. By distinguishing broadly conserved responses, such as mitochondrial fragmentation, from more stress-dependent phenotypes, such as GFP-HDEL redistribution, acetic-acid-associated vacuolar remodeling, ESCRT marker divergence, GFP-Atg8 remodeling, and stress-associated protein puncta, this study provides a descriptive map for future mechanistic testing.

One limitation of the present study is that most conclusions are based on marker localization and morphology rather than orthogonal functional or biochemical validation. In addition, not all observed phenotypes were quantified across all marker sets, and some probes may be influenced by pH or tag-dependent behavior in specific compartments. Although selected biochemical or functional readouts were included, including GFP-HDEL immunoblotting, FM4-64 pulse-chase imaging, TAG quantification, and GFP-Atg8 cleavage assays, these assays do not fully resolve the causal mechanisms underlying each phenotype. Future studies combining live-cell imaging, spatial proteomics, genetic perturbation, cargo trafficking assays, organelle contact-site analysis, and quantitative stress-survival measurements will be needed to determine how the observed remodeling events contribute to adaptation or cell fate.

Finally, our results also support a more cautious use of apoptosis-related terminology in budding yeast. Although yeast has provided powerful models for dissecting cell death-associated phenotypes, it lacks canonical mammalian Bcl-2 family architecture, and direct one-to-one extrapolation from mammalian apoptosis pathways is not always warranted (Manon, 2022). Accordingly, the strongest conclusion supported by the current work is that distinct environmental insults trigger reproducible but non-identical programs of organelle remodeling in budding yeast. These findings establish a descriptive and hypothesis-generating framework for future studies of organelle communication, stress adaptation, and cell-death-associated phenotypes in yeast, but they should not be interpreted on their own as direct evidence for a specific apoptotic or necrotic pathway.

## CRediT authorship contribution statement

Peng Sheng performed the additional experiments required for revision, conducted data analysis, and prepared the revised figures. Mengna Sun and Xinyue Wang performed the initial experiments, analyzed the data, and prepared the original figures. Lizhe Bai contributed to data analysis, assisted with figure preparation, and critically revised the manuscript. Hong Cao and Dan Li conceived and supervised the study, acquired funding, and administered the project. Dan Li drafted and revised the manuscript. All authors reviewed and approved the final version of the manuscript.

## Funding

This work was supported by the National Natural Science Foundation of China (32101033), the Key Research Project of the Health Commission of Jiangsu Province (K2023004), and the “Shuangbai Talents” Research Program of the Wuxi Health Commission (HB2023061).

## CRediT authorship contribution statement

**Peng Sheng:** Investigation, Data curation, Formal analysis, Validation, Visualization, Writing – original draft. **Mengna Sun:** Investigation, Data curation, Formal analysis, Validation, Visualization. **Xinyue Wang:** Investigation, Data curation, Formal analysis, Validation, Visualization. **Lizhe Bai:** Data curation, Formal analysis, Validation, Visualization. **Hong Cao:** Project administration, Resources, Supervision. **Dan Li:** Conceptualization, Formal analysis, Funding acquisition, Investigation, Methodology, Project administration, Supervision, Writing – review & editing.

## Declaration of competing interest

The authors declare the following financial interests/personal relationships which may be considered as potential competing interests: DanLi reports financial support was provided by National Natural Science Foundation of China (32101033). DanLi reports financial support was provided by the Key Research Project of the Health Commission of Jiangsu Province (K2023004). DanLi reports financial support was provided by the Shuangbai Talents Research Program of the Wuxi Health Commission (HB2023061). If there are other authors, they declare that they have no known competing financial interests or personal relationships that could have appeared to influence the work reported in this paper.

## References

[bib0045] Azmi I., Davies B., Dimaano C., Payne J., Eckert D., Babst M., Katzmann D.J. (2006). Recycling of ESCRTs by the AAA-ATPase Vps4 is regulated by a conserved VSL region in Vta1. J. Cell Biol..

[bib0044] Babst M., Katzmann D.J., Snyder W.B., Wendland B., Emr S.D. (2002). Endosome-associated complex, ESCRT-III, contains two functionally distinct subcomplexes. Dev. Cell.

[bib0011] Bartolutti C. (2025). UPR deficiency leads to poor growth, aneuploidy, and a trade-off between ER and general proteostasis in yeast. Genes Dev..

[bib0018] Behnia R., Panic B., Whyte J.R.C., Munro S. (2004). Targeting of the Arf-like GTPase Arl3p to the Golgi requires N-terminal acetylation and the membrane protein Sys1p. Nat. Cell Biol..

[bib0021] Birschmann I., Rosenkranz K., Erdmann R., Kunau W.H. (2005). Structural and functional analysis of the interaction of the AAA-peroxins Pex1p and Pex6p. FEBS J..

[bib0026] Castro I.G., Shortill S.P., Dziurdzik S.K., Cadou A., Ganesan S., Sargsyan A. (2022). Systematic analysis of membrane contact sites in *Saccharomyces cerevisiae* uncovers modulators of cellular lipid distribution. Elife.

[bib0008] Cebulski J., Malouin J., Pinches N., Cascio V., Austriaco N. (2011). Yeast Bax inhibitor, Bxi1p, is an ER-localized protein that links the unfolded protein response and programmed cell death in *Saccharomyces cerevisiae*. PLoS One.

[bib0014] Chaves S.R., Rego A., Martins V.M., Santos-Pereira C., Sousa M.J. (2021). Regulation of cell death induced by acetic acid in yeasts. Front. Cell Dev. Biol..

[bib0042] Chong Y.T., Koh J.L.Y., Friesen H., Duffy S.K., Cox M.J., Moses A., Moffat J., Boone C., Andrews B.J. (2015). Yeast proteome dynamics from single cell imaging and automated analysis. Cell.

[bib0035] Dea A., Pincus D. (2024). The heat shock response as a condensate cascade. J. Mol. Biol..

[bib0017] Dean N., Zhang Y.B., Poster J.B. (1997). The VRG4 gene is required for GDP-mannose transport into the lumen of the Golgi in the yeast, *Saccharomyces cerevisiae*. J. Biol. Chem..

[bib0036] Demeshkina N.A., Ferré-D’Amaré A.R. (2025). Large-scale purifications reveal yeast and human stress granule cores are heterogeneous particles with complex transcriptomes and proteomes. Cell Rep..

[bib0028] Elbaz-Alon Y., Rosenfeld-Gur E., Shinder V., Futerman A.H., Geiger T., Schuldiner M. (2014). A dynamic interface between vacuoles and mitochondria in yeast. Dev. Cell.

[bib0020] Franzusoff A., Lauze E., Howell K.E. (1992). Immuno-isolation of Sec7p-coated transport vesicles from the yeast secretory pathway. Nature.

[bib0005] Galluzzi L., Bravo-San Pedro J.M., Kroemer G. (2014). Organelle-specific initiation of cell death. Nat. Cell Biol..

[bib0006] Guaragnella N., Zdralević M., Antonacci L., Passarella S., Marra E., Giannattasio S. (2012). The role of mitochondria in yeast programmed cell death. Front. Oncol..

[bib0031] Hariri H., Ugrankar R., Liu Y., Henne W.M. (2016). Inter-organelle ER–endolysosomal contact sites in metabolism and disease across evolution. Commun. Integr. Biol..

[bib0030] Hariri H., Rogers S., Ugrankar R., Liu Y.L., Feathers J.R., Henne W.M. (2018). Lipid droplet biogenesis is spatially coordinated at ER–vacuole contacts under nutritional stress. EMBO Rep..

[bib0003] Kocik R.A., Cytrynbaum E.G., Ahrens J.M., McClean M.N., Gasch A.P. (2026). The importance of regulated resource reallocation during dynamic environmental shifts in yeast. EMBO J..

[bib0010] Korennykh A.V., Egea P.F., Korostelev A.A., Finer-Moore J., Zhang C., Shokat K.M., Stroud R.M., Walter P. (2009). The unfolded protein response signals through high-order assembly of Ire1. Nature.

[bib0027] Kornmann B., Currie E., Collins S.R., Schuldiner M., Nunnari J., Weissman J.S., Walter P. (2009). An ER–mitochondria tethering complex revealed by a synthetic biology screen. Science (1979).

[bib0032] Lam D.K., Sherlock G. (2023). Yca1 metacaspase: diverse functions determine how yeast live and let die. FEMS Yeast Res..

[bib0043] Li D. (2020). Excess diacylglycerol at the endoplasmic reticulum disrupts endomembrane homeostasis and autophagy. BMC Biol..

[bib0024] Li T., Li X., Li M., Yuan T., Ma C. (2026). Atg18 facilitates autophagosome formation via its Atg8-interacting motif in *Saccharomyces cerevisiae*. FEBS J..

[bib0001] Lin N., Xu Y., Yu X. (2022). Overview of yeast environmental stress response pathways and the development of tolerant yeasts. Syst. Microbiol. Biomanufacturing.

[bib0038] Liu C.Y., Zhu J., Xie Z. (2022). Visualizing yeast organelles with fluorescent protein markers. J. Vis. Exp..

[bib0015] Ludovico P., Sousa M.J., Silva M.T., Leão C., Côrte-Real M. (2001). *Saccharomyces cerevisiae* commits to a programmed cell death process in response to acetic acid. Microbiology (N. Y).

[bib0047] Manon S. (2022). Yeast as a tool to decipher the molecular mechanisms underlying the functions of Bcl-2 family. Explor. Target. Anti-Tumor Ther..

[bib0041] Mattiazzi Usaj M., Sahin N., Friesen H., Pons C., Usaj M., Masinas M.P.D. (2020). Systematic genetics and single-cell imaging reveal widespread morphological pleiotropy and cell-to-cell variability. Mol. Syst. Biol..

[bib0012] Mori K. (2009). Signalling pathways in the unfolded protein response: development from yeast to mammals. J. Biochem..

[bib0022] Nair U., Yen W.L., Mari M., Cao Y., Xie Z., Baba M., Reggiori F., Klionsky D.J. (2012). A role for Atg8–PE deconjugation in autophagosome biogenesis. Autophagy.

[bib0007] Ott M., Gogvadze V., Orrenius S., Mitochondria Z.B. (2007). oxidative stress and cell death. Apoptosis.

[bib0029] Pan X., Roberts P., Chen Y., Kvam E., Shulga N., Huang K., Lemmon S., Goldfarb D.S. (2000). Nucleus–vacuole junctions in *Saccharomyces cerevisiae* are formed through the direct interaction of Vac8p with Nvj1p. Mol. Biol. Cell.

[bib0025] Phillips M.J., Voeltz G.K. (2016). Structure and function of ER membrane contact sites with other organelles. Nat. Rev. Mol. Cell Biol..

[bib0040] Platzek A., Odehnalová K., Schessner J.P., Borner G.H.H., Schuck S. (2025). Dynamic organellar mapping in yeast reveals extensive protein localization changes during ER stress. Nat. Commun..

[bib0039] Rines D.R., Thomann D., Dorn J.F., Goodwin P., Sorger P.K. (2011). Live cell imaging of yeast. Methods Enzymol..

[bib0034] Rinnerthaler M., Büttner S., Laun P., Heeren G., Felder T.K., Klinger H. (2013). Mmi1, the yeast homologue of mammalian TCTP, associates with stress granules in heat-shocked cells and modulates proteasome activity. PLoS One.

[bib0016] Saravanan K., Kane P.M. (2026). Early lipid-mediated responses to hyperosmotic stress at the yeast vacuole. Mol. Biol. Cell.

[bib0004] Tartik M. (2026). Yeast stress response to synthetic constructs. ACS Synth. Biol..

[bib0013] Townsley F.M., Frigerio G., Pelham H.R.B. (1994). Retrieval of HDEL proteins is required for growth of yeast cells. J. Cell Biol..

[bib0019] Trautwein M., Schindler C., Gauss R., Dengjel J., Hartmann E., Spang A. (2006). Arf1p, Chs5p and the ChAPs are required for export of specialized cargo from the Golgi. EMBO J..

[bib0009] Welihinda A.A., Kaufman R.J. (1996). The unfolded protein response pathway in *Saccharomyces cerevisiae*: oligomerization and trans-phosphorylation of Ire1p (Ern1p) are required for kinase activation. J. Biol. Chem..

[bib0033] Wissing S., Ludovico P., Herker E., Büttner S., Engelhardt S.M., Decker T., Link A., Proksch A., Rodrigues F., Côrte-Real M., Fröhlich K.U., Manns J., Candé C., Sigrist S.J., Kroemer G., Madeo F. (2004). An AIF orthologue regulates apoptosis in yeast. J. Cell Biol..

[bib0046] Yoo H., Bard J.A.M., Pilipenko E.V., Drummond D.A. (2022). Chaperones directly and efficiently disperse stress-triggered biomolecular condensates. Mol. Cell.

[bib0002] Yuan B. (2024). Regulatory mechanisms underlying yeast chemical stress response and development of robust strains for bioproduction. Curr. Opin. Biotechnol..

[bib0023] Zhang T., Lin Y., Zhang Z., Wang Z., Zeng F., Wang Q. (2025). Roles and applications of autophagy in guarding against environmental stress and DNA damage in *Saccharomyces cerevisiae*. FEBS J..

[bib0037] Zhu J., Zhang Z.T., Tang S.W., Zhao B.S., Li H., Song J.Z., Li D., Xie Z. (2019). A validated set of fluorescent-protein-based markers for major organelles in yeast (*Saccharomyces cerevisiae*). mBio.

